# Active Impedance Control of Bioinspired Motion Robotic Manipulators: An Overview

**DOI:** 10.1155/2018/8203054

**Published:** 2018-10-18

**Authors:** Hayder F. N. Al-Shuka, Steffen Leonhardt, Wen-Hong Zhu, Rui Song, Chao Ding, Yibin Li

**Affiliations:** ^1^School of Control Science and Engineering, Shandong University, Jinan, China; ^2^Philips Chair for Medical Information Technology (MedIT), Helmholtz Institute, RWTH Aachen University, Aachen, Germany; ^3^Canadian Space Agency, Longueuil, Canada

## Abstract

There are two main categories of force control schemes: hybrid position-force control and impedance control. However, the former does not take into account the dynamic interaction between the robot's end effector and the environment. In contrast, impedance control includes regulation and stabilization of robot motion by creating a mathematical relationship between the interaction forces and the reference trajectories. It involves an energetic pair of a flow and an effort, instead of controlling a single position or a force. A mass-spring-damper impedance filter is generally used for safe interaction purposes. Tuning the parameters of the impedance filter is important and, if an unsuitable strategy is used, this can lead to unstable contact. Humans, however, have exceptionally effective control systems with advanced biological actuators. An individual can manipulate muscle stiffness to comply with the interaction forces. Accordingly, the parameters of the impedance filter should be time varying rather than value constant in order to match human behavior during interaction tasks. Therefore, this paper presents an overview of impedance control strategies including standard and extended control schemes. Standard controllers cover impedance and admittance architectures. Extended control schemes include admittance control with force tracking, variable impedance control, and impedance control of flexible joints. The categories of impedance control and their features and limitations are well introduced. Attention is paid to variable impedance control while considering the possible control schemes, the performance, stability, and the integration of constant compliant elements with the host robot.

## 1. Introduction

When a robot is in contact with the environment via its end effector, some important points should be noted:

- Given a specific degree of freedom, it is not possible to independently regulate the position and the contact force. For example, if the task of the target robot is to write something, neglecting control of the interaction force may lead to either loss of contact or hard pressure on the target environment [1]. In general, for rigid or dynamic interaction environments, pure position control schemes are not recommended, especially if the environment is stiff; the contact forces may reach unsafe values [2]
- In addition, the robot loses some degrees of freedom (DoFs) during the contact phase. Consequently, the generalized coordinates of the target robot might be larger than its DoFs due to its constrained motion; this constitutes a closed-chain mechanism with redundant coordinates [3]
- The robot may change its configuration during a transition from an open-chain mechanism to a closed-chain mechanism. In effect, three motion phases can be produced: the free motion phase, the contact motion phase (impact phase), and the constrained motion phase. Each phase can have its own features and control law [3]

One of the solutions to regulate and control the interaction forces is hybrid position/force control proposed by Raibert and Craig [4]. The hybrid force-position control decouples the task space into position-controlled space and force-controlled space. Then the hybrid position/force control law is designed to track the desired position and force references. However, this scheme does not take into consideration the impedance effect between the environment and the robot end effector.

In effect, impedance control plays an important role in any workspace that involves human-robot interactions. The idea behind it is to control the mechanical impedance of a host robot regulating the interaction forces produced by the coupling between the robot and the environment dynamics; mechanical impedance can be defined as the ratio of the output force to the input velocity (motion). For linear systems, mechanical admittance is the inverse of mechanical impedance; it can be defined as the ratio of input velocity (motion) to the output force. In general, the robot can ideally behave as an impedance and the contact environment is an admittance; however, this could not be the case for multibody robotic systems with heavy links and actuators [5, 6]. Impedance control is inspired by the human behavior during contact with different environments. Humans have a considerable amount of adaptability to change muscle impedance (e.g., stiffness) when in contact with an unknown environment. If the environment is stiff, the robot should be soft and vice versa. Rigid robots, however, do not have this capability; in principle, they are stiff. They are well suited for precise free motion space, but problems can occur when moving in an unstructured environment. Excessive interaction forces should be avoided. This can be achieved by making the robots change their stiffness. Therefore, Hogan proposed active impedance control which is based on the biomechanics of human motion in free and constrained spaces [5, 6]. The idea behind impedance control is to design a user-defined dynamic relationship between the reference trajectory of the end effector and the interaction contact force/torque along each axis. However, a trade-off occurs between the tracking of the position and the interaction forces [7]. Hogan proposed two models of impedance control [5, 6]: torque- or force-based impedance control and position-based impedance control. Due to the related limitations of conventional impedance control, Anderson and Spong [8] proposed to use hybrid impedance control. The idea is to exploit the concept of hybrid position/force control and integrate it with impedance control. A robust control version of hybrid impedance control with inner acceleration was proposed by Liu and Goldenberg [9] such that the convergence of position, velocity, and acceleration is proven with bounded force error. Zhu et al. [10] made a link between hybrid control and impedance control using virtual decomposition control for two six-joint industrial robots. Extensions of other schemes were proposed for force tracking impedance control [11–18]. Force tracking impedance control has led researchers towards variable impedance control, which means that the target impedance parameters change adaptively for safe interaction motion with an unknown environment. This behavior is similar to that of humans, who have adaptable flexibility and impedance to deal with interaction tasks. The challenge of variable impedance control may lie in how the analyst can select the impedance time-varying parameters with guaranteed stability. The same problem might be encountered when dealing with fixed impedance parameters [7, 19]. The limitations inherent in active impedance control resulted in the development of new generations of actuator technology, ranging from series elastic actuators to variable impedance actuators [20]. These actuators include constant or variable compliant elements in their design [21, 22]. The idea behind these actuators is to imitate the behavior of human motion during contact with unknown constrained spaces. By controlling the stiffness of the target robot, the robot can adaptively comply with the interaction forces and generate safe contact. However, the way to integrate active impedance control with these passive design-based actuators is important and presents various problems related to control and stability.

**Figure 1:**
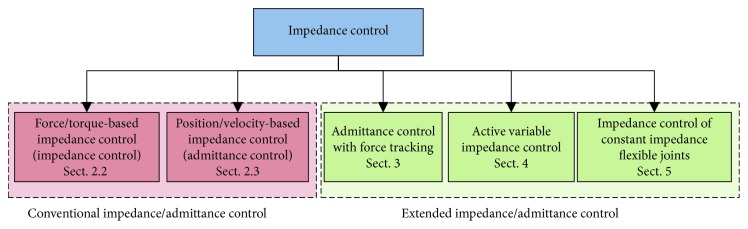
A general classification of the impedance control approaches. The paper is organized according to the depicted classification.

In view of the above, this paper is aimed at summarizing the different schemes of impedance control with miscellaneous control modes (see Figure 1). The features and disadvantages of each scheme are described. The problems of stability are discussed, and the applications of impedance control are presented.

The paper is organized as follows: Section 2 introduces the background of the two main modes of conventional impedance control.  Section 3 examines force tracking position-/velocity-based impedance control, and Section 4 discusses the scheme of variable impedance control.  Section 5 describes impedance control of flexible joints with constant and variable compliance, and Section 6 presents the conclusions.

## 2. Background

**Figure 2:**
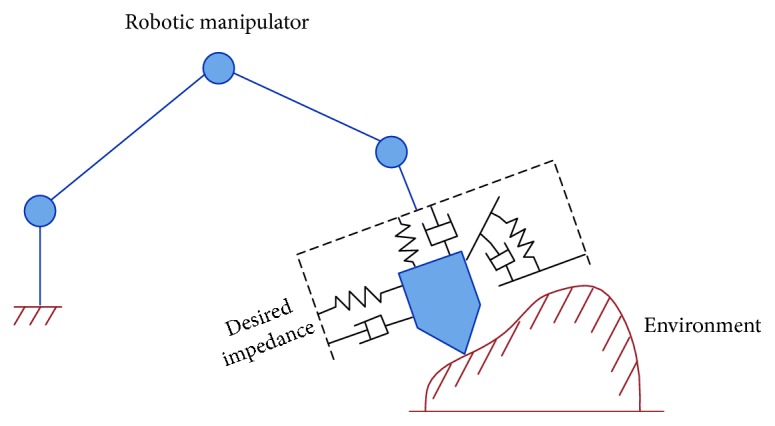
Description of impedance control for a robot in contact with the external environment [1, 32].

Although the impedance control schemes are referred to as an indirect force method, some of these schemes can include a force tracking loop with the impedance target, e.g., the position-/velocity-based impedance control (admittance control) can be modified to improve the interaction force tracking problems (this is discussed in the following sections). As mentioned, the idea of impedance or admittance control is to generate a dynamic relationship between the interaction force/torque and the position/velocity trajectory of the robot end effector by using the virtual *mass-spring-damper* system; see Figure 2 for a description of the idea of impedance control.

By tuning the parameters of the impedance parameters, a suitable performance can be obtained for the host robot; there is a deviation in robot motion associated with and coupled with the deviation of interaction force. Basically, impedance control may consist of two nested control loops: an outer impedance control loop and an inner position/velocity/force control loop. For more details on the interaction force control schemes, see [23, 24]. Below, we present the modeling of robots with interaction forces and how to obtain a suitable formulation of impedance control for redundant and nonredundant robots. Then the two conventional categories of impedance control are described.

**Remark 1 .**

The three related subcategories of impedance control are stiffness control [1], compliance control [25], and damping control [26]; for details, see [1, 25, 26].

### 2.1. Dynamic Modeling of Robots in Constrained Motion

Target impedance dynamics (outer impedance loop) is preferably expressed in terms of the task coordinate frames, since the task geometry may decide which directions are motion constrained and force sensitive [7]. The task specifications for motion and interaction forces and the force feedback are closely related to the end effector. A description of the dynamic behavior of the end effector and its association with the external environment is essential for high-performance manipulator control [27]. In general, impedance control consists of two control loops: an outer impedance loop regulating the interaction between the end effector and the external environment and an inner control loop which can be a torque control loop or a position/velocity control loop. For an outer impedance loop, representation of the dynamics of the impedance target in terms of task space is necessary. For the inner control loop, there are two possibilities of coordinate representation for the control law. For force-based impedance control, the inner joint space torque control requires a transformation of the commanded forces generated by the outer loop into a commanded torque that should be tracked. Accordingly, this requires calculation of the Jacobian online. For the position-/velocity-based impedance control (admittance control), the inner position/velocity control can be represented in joint space by transformation of the commanded task coordinates into joint coordinates using inverse kinematics [28–30]. However, the inner position/velocity control law can be represented in task coordinates, as presented in [31]. In effect, despite the usefulness of task space formulation for implementation of high-performance control schemes, measurement of the end effector position and orientation (without the use of geometric Jacobian) is not easy; this may require vision technology. On the other hand, implementation of joint space control combined with a Cartesian impedance outer loop may require calculation of Jacobians and inverse kinematic schemes, which could be computationally complex.

Consider an *n* − DoF robot operating in m-dimensional Cartesian coordinates. The kinematics of the host robot can be expressed as

(1) $$\begin{array}{c} x=T\left(q\right), \end{array}$$

(2) $$\begin{array}{c} \dot{x}=J\left(q\right)\dot{q}, \end{array}$$

where *x* ∈ ℝ^*m*^ is the Cartesian position of the end effector, *T*(.): ℝ^*n*^⟶ℝ^*m*^ denotes to the forward kinematics, *q* ∈ ℝ^*n*^ is the joint position, and *J*(.) ∈ ℝ^*m*×*n*^ is the manipulator Jacobian matrix. For robots in constrained space, the 2^nd^ Lagrangian formulation can be used for modeling. Thus, the dynamic equation of rigid joint fully actuated robots can be expressed in joint space as follows.

(3) $$\begin{array}{c} M\left(q\right)\;\ddot{q}+C\left(q,\dot{q}\right)\dot{q}+g\left(q\right)=J{\left(q\right)}^{T}f_{e}+\tau_{l},\\ I_{m}\ddot{\theta }+\tau_{f}\left(\dot{\theta }\right)+G\tau_{l}=\tau_{m},\\ L\dot{\iota }+R\iota +K_{b}\dot{\theta }=u, \end{array}$$

where *M*(.) ∈ ℝ^*n*×*n*^ represents the inertia matrix, *C*(.) ∈ ℝ^*n*×*n*^ is the Coriolis and centripetal matrix, *g*(.) ∈ ℝ^*n*^ is the gravity, *f*_*e*_ ∈ ℝ^*m*^ denotes the external and interactional forces, *τ*_*l*_ ∈ ℝ^*n*^ is the output torque on the link side, *I*_*m*_ ∈ ℝ^*n*×*n*^ is the equivalent inertia matrix on the motor side, *θ* ∈ ℝ^*n*^ is the motor angular position vector, *τ*_*f*_ ∈ ℝ^*n*^ is the friction vector, *G* ∈ ℝ^*n*×*n*^ refers to the diagonal gear ratio matrix, *τ*_*m*_ ∈ ℝ^*n*^ is the motor torque that is equal to *K*_*τ*_*ι*, with *K*_*τ*_ ∈ ℝ^*n*×*n*^ being denoted to the torque constant and *ι* ∈ ℝ^*n*^ is the armature current, *L* ∈ ℝ^*n*×*n*^ is the diagonal inductance matrix, *R* ∈ ℝ^*n*×*n*^ is the resistance matrix, *K*_*b*_ ∈ ℝ^*n*×*n*^ is the diagonal EMF constant matrix, and *u* ∈ ℝ^*n*^ is the input voltage control.

In general, the following points should be noted:

- For a robot having (*m* = *n*), i.e., the number of the generalized coordinates is equal to the task space coordinates, the robot is nonredundant. Whereas if *n* > *m*, i.e., the number of the generalized coordinates is higher than the task space coordinates, the robot is called a “kinematically redundant” robot with redundant coordinate *r* = *n* − *m*
- In general, there are two possible aspects of redundancy problems, i.e., motion redundancy and torque redundancy [32–34]. For better performance of redundant robots, the null space dynamics should be considered, i.e., dealing only with task space impedance control may not be sufficient. For more details on redundant robots and modified impedance control, see [35–37]
- Equation (3) can be transformed into task space coordinates by using the kinematic relationships of (2). For more details on task space dynamics considering fully actuated, underactuated, and overactuated robotic systems, the reader is referred to [27, 28, 38, 39]
- Consideration of actuator dynamics is important for a robot with high-velocity movement and highly varying loads. For more details on the effect of neglecting actuator dynamics, the reader is referred to [31]. In addition, Zhu [40] has proposed three motor control modes: the torque control mode, the current control mode, and the voltage control mode. An electric motor can be in the motor torque control mode when the armature current is well controlled by a current servo amplifier and the motor torque/current constant is known. Otherwise, an electric motor should be in the motor current control mode when only the armature current is well controlled but the torque/current constant is unknown. Finally, an electric motor must be in the motor voltage control mode when no current servo control is available
- On the other hand, (3) is concerned with electrically driven robots; for dynamic modeling of hydraulic and pneumatic actuators, see [40–42]
- Section 5 will consider the effect of joint flexibility and the associated control problems

### 2.2. Force-/Torque-Based Impedance Control

**Figure 3:**
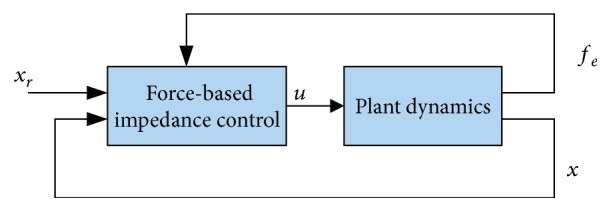
Generic diagram of force-/torque-based impedance control [29, 30]. For the force-/torque-based impedance control, an inner feedback loop for force/torque is optional; see, e.g., (6) that does not use an inner force control loop. For explicit nested loops, the reader is referred to [30].

The idea behind force-based impedance control (simply called “impedance control” in the literature) is to make the controller react to the motion deviation by generating forces [2, 28]. It consists of two control loops: an outer position loop (target impedance filter) and an optional inner force loop. The controller may attempt to stiffen a soft force source [28]; see Figure 3 for a generic description of force- (torque-) based impedance control.

**Figure 4:**
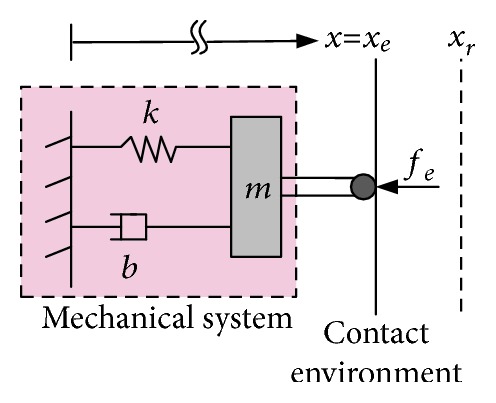
System dynamics in contact with the external environment [62]. The coordinate *x*_*e*_ represents the position of the environment, and the coordinate *x*_*r*_ represents the reference equilibrium trajectory that should slightly be making inside the contact environment to maintain contact. The position error is equal to the difference between the actual position (*x*) and the reference (*x*_*r*_); this error should guarantee a compliant contact with the environment.

To motivate the concept of impedance control, consider the following simple second-order system (Figure 4):

(4) $$\begin{array}{c} m\ddot{x}+b\dot{x}+kx=u+f_{e}, \end{array}$$

where *x* refers to the position of the system mass (*m*), *b* represents the damping coefficients, *k* denotes the system stiffness, *u* is the input control, and *f*_*e*_ is the external force affecting the system (it can be the interaction contact force or any external force).

As stated, impedance control attempts to make a dynamic relationship between the interaction force and position error by assuming a virtual mass-spring-damper model with the desired trajectory; accordingly, the target impedance function can be expressed as [1]

(5) $$\begin{array}{c} m_{d}\left(\ddot{x}-{\ddot{x}}_{r}\right)+b_{d}\left(\dot{x}-{\dot{x}}_{r}\right)+k_{d}\left(x-x_{r}\right)=f_{e}, \end{array}$$

where *m*_*d*_, *b*_*d*_, and *k*_*d*_ are the desired target impedance coefficients that govern the performance of the controller.

Changing the structure of the target impedance dynamics or the behavior of the target impedance coefficients leads to different impedance control strategies. Substituting (5) into (4) may lead to the following closed-loop control system

(6) $$\begin{array}{c} u=\left(b-{mm}_{d}^{-1}b_{d}\right)\dot{x}+\left(k-{mm}_{d}^{-1}k_{d}\right)x-\left(1-{mm}_{d}^{-1}\right)f_{e}+{mm}_{d}^{-1}\left(b_{d}{\dot{x}}_{r}+k_{d}x_{r}\right)+m{\ddot{x}}_{r}. \end{array}$$

As can be seen, the feedback controller of (6) needs the measurements of interaction force and the state variables of the end effector.

Consider the case *m* = *m*_*d*_, then (6) can be simplified to

(7) $$\begin{array}{c} u=\left(b-b_{d}\right)\dot{x}+\left(k-k_{d}\right)x+\left(b_{d}{\dot{x}}_{r}+k_{d}x_{r}\right)+m{\ddot{x}}_{r}, \end{array}$$

which represents classical velocity and position feedback control with a feed-forward term denoted by the desired acceleration. Equation (7) is a special case of (6); however, the impedance implementations are different in terms of stability and transparency. There are several passivity results for the control law (7) including the case of time-varying target impedance (see, e.g., [43] and the references therein). In effect, placing a force sensor at the robot end effector can be difficult in some applications such as robotic surgery, and hence, (7) is preferable.

In effect, there are three possible models for representing the target impedance dynamics that correlate the dynamic relationship between the position and contact forces

(8) $$\begin{array}{c} m_{d}\left(\ddot{x}-{\ddot{x}}_{r}\right)+b_{d}\left(\dot{x}-{\dot{x}}_{r}\right)+k_{d}\left(x-x_{r}\right)=f_{e}, \end{array}$$

(9) $$\begin{array}{c} m_{d}\left(\ddot{x}\right)+b_{d}\left(\dot{x}-{\dot{x}}_{r}\right)+k_{d}\left(x-x_{r}\right)=f_{e}, \end{array}$$

(10) $$\begin{array}{c} m_{d}\left(\ddot{x}\right)+b_{d}\left(\dot{x}\right)+k_{d}\left(x-x_{r}\right)=f_{e}. \end{array}$$

Equations (8), (9), and (10) is essentially the same; only the reference signal has different components. Besides, (8)–(10) makes a compromise between the position and the contact forces such that there could be deviations in the desired position and the force references. Since a control loop based on force error is missing, forces are only indirectly assigned by controlling position. The choice of a specific stiffness in the impedance model along a Cartesian direction results in a trade-off between contact forces and position accuracy in that direction. The effect of virtual impedance parameters on the system response can be investigated by manipulating one of the impedance parameters and fixing the others. Accordingly, larger virtual mass can lead to slow response and vice versa, whereas the virtual stiffness is responsible for the response attenuation. The advantage of damping coefficient *b*_*d*_ is to shape the transient response. See [44] for more details on the parameter tuning. As a rule of thumb, the stiffer the environment is, the softer is the impedance stiffness *k*_*d*_. The external environment force can be eliminated by substituting (5) into (4) such that the contact force is neglected; however, in this case, the measurement of acceleration is required, which is very noisy.

Seraji and Colbaugh [45] used both (8) and (10) to derive the equations of the steady-state force and position errors, respectively, whereas Yoshikawa [1] used (9) to derive impedance control for both free and constrained spaces. Yoshikawa demonstrated that when there is no contact force, the controller represents position and velocity feedback control. Huang and Chien [31] used regressor-free adaptive backstepping control for flexible joints; they used (8) as the target impedance dynamics. Khan et al. [46] used adaptive impedance control based on the target impedance dynamics of (8) for an upper limb assist exoskeleton. Please see [7, 47, 48] for more details on the characteristics and limitations of this impedance control scheme.

### 2.3. Position-/Velocity-Based Impedance Control (Admittance Control)

**Figure 5:**
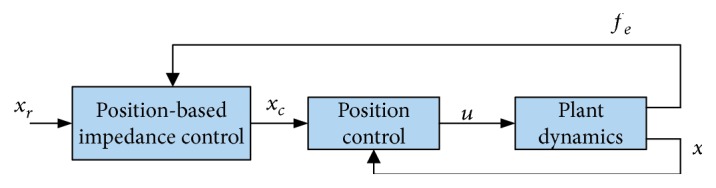
Schematic diagram of position-based impedance control [29, 30].

In admittance control, the controller aims to soften the stiff position source via reacting to the interaction forces by imposing deviation from the desired motion [2, 28]. Position-/velocity-based impedance control consists of two control loops: an inner position/velocity loop to control the compliant position/velocity references and an outer loop to provide the desired target impedance dynamics that delivers the commanded compliant references (see Figure 5 for a general description).

Below is a simple motivating example that describes the position-based impedance control. However, for the velocity-based impedance control, a similar strategy can be used by replacing the desired and commanded position references with the velocity reference.

**Figure 6:**
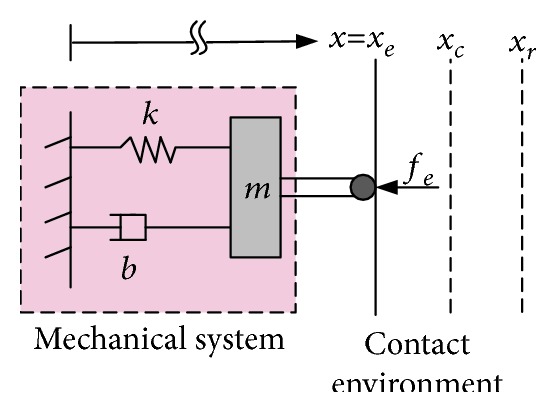
System dynamics in contact with the external environment. The philosophy of impedance target dynamics changes due to adding the commanded impedance trajectory *x*_*c*_ [62].

For the position-based impedance control of the previous 2^nd^-order system described in (4), (8)–(10) should be modified to isolate the inner position control loop from the outer impedance control loop. This can be done by introducing a new variable called “the commanded impedance reference trajectory” *x*_*c*_, for the end effector which results from the desired references of the end effector and measurement of the interaction force wrench (see Figure 6 for details). Accordingly, the outer impedance filter can be expressed as [49–54]

(11) $$\begin{array}{c} m_{d}\left({\ddot{x}}_{c}-{\ddot{x}}_{r}\right)+b_{d}\left({\dot{x}}_{c}-{\dot{x}}_{r}\right)+k_{d}\left(x_{c}-x_{r}\right)=f_{e}, \end{array}$$

(12) $$\begin{array}{c} m_{d}\left({\ddot{x}}_{c}\right)+b_{d}\left({\dot{x}}_{c}-{\dot{x}}_{r}\right)+k_{d}\left(x_{c}-x_{r}\right)=f_{e}, \end{array}$$

(13) $$\begin{array}{c} m_{d}\left({\ddot{x}}_{c}\right)+b_{d}\left({\dot{x}}_{c}\right)+k_{d}\left(x_{c}-x_{r}\right)=f_{e}, \end{array}$$

The inner position control can be implemented using the proportional-integral-derivative (PID) family in our simple example; thus, the control law can be expressed as

(14) $$\begin{array}{c} u=k_{p}\left(x_{c}-x\right)+k_{v}\left({\dot{x}}_{c}-\dot{x}\right), \end{array}$$

where *k*_*p*_ and *k*_*v*_ are the feedback gains.

In effect, the well-known nonlinear schemes, e.g., feedback linearization control (computed torque control), passivity-based control, robust sliding mode control, and mode reference adaptive control, can be used for the inner position control loops [31, 49–55].

### 2.4. Position-Based Impedance Control vs. Force-Based Impedance Control

In effect, force-based impedance control and position-/velocity-based impedance control are based on the assumption of a force-controlled system and a position-controlled system; therefore, their performance and stabilities may differ [48]. Some important points need to be considered when using impedance control [28, 32, 48]:

- For the force-/torque-based impedance control, an inner feedback loop for force/torque is optional while for position-based impedance control, the inner position loop is required
- Since most of the industrial electromechanical manipulators are equipped with servo position control loops, position-/velocity-based impedance control might avoid redesigning the inner position loop
- For the desired stiff impedance behavior, force-based impedance control may encounter instability problems due to the amplification of noise. If the environment is soft (compliant), the stiffness of the end effector should be stiffer and vice versa. Accordingly, force-based impedance control might be suitable for interaction with a stiff environment. In contrast, position-based impedance control is more suitable to implement stiff behavior than compliant behavior, i.e., it is suitable for interaction with a compliant environment
- The performance and stability of force-based impedance control may depend on back drivability and the amount of friction for the host system, whereas the performance of position-based impedance control can depend on the performance of inner position control and the quality of the force measurement

For more details on the differences among these categories of impedance control, see [56].

### 2.5. Position-Based Impedance Control vs. Velocity-Based Impedance Control

Literature proves that an inner velocity control loop can improve the performance and the stability problems associated with impedance control. However, the following points should be considered:

- Using a force loop around a position loop seems to be very natural and, therefore, this was exactly the mainstream approach used in the 1980s; see [40, 57–59] for example. Its main problem is the stability in rigid contact. In order to maintain the stability, the small gain theory is employed, leading to a very small gain and a very slow process. In contrast, the velocity-based force control is directly based on passivity theory to ensure the stability (using the Lyapunov-like function) and, therefore, has higher force control bandwidth; see [40, 57, 59] for more details
- Referring to (11), neglecting the stiffness term and assuming a unit step force, the inner position control loop behaves as an integrator. Whereas, the inner velocity control loop under the same conditions can behave as a first-order transfer function (low pass filter) that may not require aggressive tuning [60]. However, the inner velocity/position control can impose a constraint on lower bounds of desired impedance [56, 61]
- In some robotic applications, the desired position trajectory can be unknown, and thereby, the use of an inner velocity control loop is more suitable. Examples of these applications are the unknown final destination of human-robot cooperation [60] and the difficulties associated with determining the desired position trajectory of a low-impact docking mechanism [44]

## 3. Position-/Velocity-Based Impedance Control with Force Tracking

In humans, the stiffness of muscles plays an important role in dextrous and robust motions. For example, the human arm can control the interaction contact force by modifying its muscle stiffness such that the interaction contact force can be either increased by making the arm stiffer or decreased by reducing the arm's stiffness. In addition, an individual can keep the force tracking error within a specified range in the presence of disturbances and uncertainty [62]. In effect, the target impedance dynamics of (8)–(10) and that of (14) are asymptotically stable in free space, while there are steady-state position and force errors in constrained space.

In general, most robotic systems need to be in contact with the external environment. Regulation of the interaction force is necessary to avoid problems related to instability and safety. Some robot applications include control and stabilization of the constant value interaction force, such as with deburring, welding, and grinding [63]. Nevertheless, human-robot interaction applications require time-variant interaction forces, such as robot-aided cell injection [64, 65] and rehabilitation applications [66, 67]. Accordingly, conventional impedance control may not be suitable for these applications and large deviations of position and forces might be produced; tracking of the time-varying force control combined with impedance behavior is required.

Accordingly, the exact position and force tracking may not occur in conventional impedance control strategies. The main limitation of impedance control is that the interaction forces are controlled indirectly by selecting the desired impedance dynamics. However, this may demand accurate knowledge of environment parameters (e.g., environment location and stiffness) which are difficult to specify in practical applications [45, 68].

To illustrate the importance of knowledge of the environment parameters, consider the following scalar target impedance function

(15) $$\begin{array}{c} m_{d}\left({\ddot{x}}_{r}-{\ddot{x}}_{c}\right)+b_{d}\left({\dot{x}}_{r}-{\dot{x}}_{c}\right)+k_{d}\left(x_{r}-x_{c}\right)=f_{r}-f_{e}, \end{array}$$

replacing the environment contact force by the difference between the required desired force (*f*_*r*_) and the sensed contact force (*f*_*e*_). This modification might be necessary for force tracking.

If the desired reference trajectory maintains constant values, their first and second derivatives are equal to zero. Thus, (15) becomes

(16) $$\begin{array}{c} -m_{d}{\ddot{x}}_{c}-b_{d}{\dot{x}}_{c}+k_{d}\left(x_{r}-x_{c}\right)=f_{r}-f_{e}. \end{array}$$

Using a simple spring model to represent the deformation of the environment (assuming that environment stiffness dominates its deformation), the interaction force can be expressed as

(17) $$\begin{array}{c} f_{e}=k_{e}\left(x-x_{e}\right). \end{array}$$

Rewriting the above equation to get the end effector position leads to

(18) $$\begin{array}{c} x=\frac{f_{e}}{k_{e}}+x_{e}. \end{array}$$

Inserting the force error in (18) leads to

(19) $$\begin{array}{c} x=\frac{\left(f_{r}-e_{f}\right)}{k_{e}}+x_{e}, \end{array}$$

with *e*_*f*_ = (*f*_*r*_ − *f*_*e*_).

Because the objective of the inner position control loop is to track the commanded compliant impedance references (*x*_*c*_), a position error can be produced. Accordingly, *x*_*c*_ can be expressed as

(20) $$\begin{array}{c} x_{c}=\frac{\left(f_{r}-e_{f}\right)}{k_{e}}+x_{e}+e_{p}, \end{array}$$

with

(21) $$\begin{array}{c} e_{p}=x_{c}-x=x_{c}-\frac{\left(f_{r}-e_{f}\right)}{k_{e}}-x_{e}. \end{array}$$

Substituting (20) in (16) produces the following force/position error differential equation (closed loop)

(22) $$\begin{array}{c} m_{d}{\ddot{e}}_{f}+b_{d}{\dot{e}}_{f}+\left(k_{d}+k_{e}\right)e_{f}=k_{e}k_{d}x_{e}+m_{d}{\ddot{f}}_{r}+b_{d}{\dot{f}}_{r}+k_{d}f_{r}+k_{e}\left(m_{d}{\ddot{e}}_{p}+b_{d}{\dot{e}}_{p}+k_{d}\left(e_{p}-x_{r}\right)\right). \end{array}$$

If the impedance system reaches the steady-state region assuming that the desired environment force is of constant value, the steady-state force error can be expressed as

(23) $$\begin{array}{c} e_{f}=\frac{k_{d}k_{e}}{k_{d}+k_{e}}\left[\frac{f_{r}}{k_{e}}+x_{e}+e_{p}-x_{r}\right]. \end{array}$$

Let *x*_*r*_ = *x*_*e*_ + (*f*_*r*_/*k*_*e*_) which *includes adequate knowledge of the environment parameters*; then (23) reduces to the following equation

(24) $$\begin{array}{c} e_{f}=\frac{k_{d}k_{e}}{k_{d}+k_{e}}e_{p}, \end{array}$$

in which *the position error plays an important role in the steady-state force error*.

Accordingly, the convergence of interaction force tracking could not be ensured in position-based impedance control, especially with uncertain environment stiffness and uncertain modeling of the host robotic system [69]. Three essential techniques are available to attenuate the force tracking error: (i) modification of the reference trajectory combined with estimation of the environment geometry and physics [45], (ii) modification of the target stiffness to carefully control the required interaction force [62], and (iii) modification of both the reference trajectory and the target stiffness [69].

Seraji and Colbaugh [45] proposed two control schemes for position-based impedance control with contact force tracking. The key idea of the schemes is to modify the desired reference trajectory required to compensate for the environment force error considering uncertain environment stiffness and location. The first scheme used the model reference adaptive control (MRAC) to generate the desired position reference online as a function of the environment force error. The second scheme is designed based on the indirect adaptive control such that the environment parameters (stiffness and location) are estimated online and desired position references are generated based on these estimates; further detailed results on indirect adaptive control are described in [70]. Lee and Buss [62] preferred to change the target (virtual) stiffness for environment force tracking because modification of the desired reference trajectory is unintuitive and the small change of *x*_*r*_ may lead to drastic changes in the environment forces. The target stiffness is variable and represents the PD controller of environment force error. Therefore, the impedance model has been designed as

(25) $$\begin{array}{c} m_{d}\left({\ddot{x}}_{r}-{\ddot{x}}_{c}\right)+b_{d}\left({\dot{x}}_{r}-{\dot{x}}_{c}\right)+k_{d}\left(t\right)\left(x_{r}-x_{c}\right)=f_{e}, \end{array}$$

with

(26) $$\begin{array}{c} k_{d}\left(t\right)=\frac{k_{p}e_{f}+k_{v}{\dot{e}}_{f}}{x_{r}-x_{c}}, \end{array}$$

where *k*_*p*_ and *k*_*v*_ are the proportional and derivative control gains, respectively. In effect, the stiffness effect is no longer present and it just becomes a term to correct for errors in the force tracking. Its value could be negative or time varying according to the proposition of the authors.

Kim et al. [69] used position-based impedance control for force tracking of a wall-cleaning unit. The proposed outer impedance filter includes the adaptation of the virtual stiffness accompanied by modification of the desired position references. The target impedance stiffness is variable and represents the PID controller in terms of environment force error and the model-following error. Accordingly, the proposed impedance model can be expressed exactly as (25) with the following desired virtual stiffness

(27) $$\begin{array}{c} k_{d}\left(t\right)=\frac{\left(k_{p}e_{f}+k_{v}{\dot{e}}_{f}+k_{i}\int_{0}^{t}e_{f}\left(\tau \right)d\left(\tau \right)\right)}{\left(x_{r}-x_{i}\right)}+k_{0}, \end{array}$$

(28) $$\begin{array}{c} x_{r}=x_{r}^{0}-\gamma \left(x_{r}-x_{c}\right), \end{array}$$

where *k*_*p*_, *k*_*v*_, and *k*_*i*_ denote the feedback gains, *k*_0_ denotes the initial impedance stiffness, *x*_*r*_^0^ represents the initial desired equilibrium trajectory, and *γ* is constant. The Routh-Hurwitz stability criterion was used as a basis to verify the stability of the proposed controller.

For more details on force tracking-based admittance control, see [11–18, 68]. In summary, the following points need to be considered:

- In effect, making the system stiffness variable imitate human behavior is considered to be variable impedance control (see explanation in the following section)
- The knowledge of environment stiffness and location is necessary for force tracking-based admittance control
- An inner velocity control loop can alternatively be used with features discussed in Section 2.5
- The derivative of the environment force error is required in some schemes, that is, undesirable. The two possible techniques to solve this problem are (i) making a filter for the sensed force signal then differentiating the filtered signal [71] or (i) exploiting the simple spring model for the environment [72]
- Most researchers assume that the decoupled outer impedance filter can simplify the control problem such that stability analysis and performance of the proposed force tracking impedance filter may depend on the linear control theory, such as root locus analysis and Routh-Hurwitz stability
- An important observation is that virtual stiffness of impedance behavior can lead to steady-state errors; therefore, cancelling this term may lead to zero steady-state errors [16, 73]

## 4. Variable Impedance Control

For most biological movements, muscles behave as mechanical actuators with a nonlinear stiffness behavior; according to biological studies, muscle viscosity can be considered constant. The force-velocity relationship includes nonlinear characteristics during contraction and stretching; increasing the applied force may result in an increase in muscle stiffness. It is important to note that the slopes of the impedance curve represent the muscle impedance associated with muscle movement [74, 75] and the references therein. The impedance profiles of the human joint can vary during motion [76]. In effect, humans can grasp objects softly and safely by regulating muscle stiffness. Moreover, it is known that the locomotion of humans consists of miscellaneous motion phases, e.g., a single-support phase, double-support phase, and jumping. Therefore, humans should modify muscle stiffness to attenuate any heterogeneous disturbances or even to track desired interaction forces [77].

There are essential applications that the robot is in contact with the human such as exoskeletons, orthosis, and prostheses. In view of the above statement, using the conventional impedance control with fixed coefficients, e.g., fixed stiffness, cannot achieve the required target impedance for the human-robot interaction. Accordingly, variable stiffness-based impedance control can improve the performance of the desired force tracking and the dexterity of the robotic system. It is a suitable strategy for modulation of the parameters of the impedance behavior such that stability is guaranteed and the performance is improved and safer. This policy of changing stiffness is explained above (Section 3) and is mentioned here due to its correlation with variable impedance control; see also [62, 73, 78].

Mathematically, the target impedance behavior with variable parameters can be expressed as

(29) $$\begin{array}{c} m_{d}\left(t\right)\left({\ddot{x}}_{r}-{\ddot{x}}_{c}\right)+b_{d}\left(t\right)\left({\dot{x}}_{r}-{\dot{x}}_{c}\right)+k_{d}\left(t\right)\left(x_{r}-x_{c}\right)=f_{r}-f_{e}, \end{array}$$

with possibly time-varying *m*_*d*_(*t*), *b*_*d*_(*t*), and *k*_*d*_(*t*).

In view of the above statement, there are two main objectives for equipping the target impedance with variable impedance:

- to track interaction force references; please see Section 3
- to increase adaptability and to imitate the biological behavior during contact with different environment stiffness

However, straightforward implementation of impedance control with time-varying virtual impedance parameters can destroy passivity conditions of the system unless a proper impedance model is selected. To prove this, consider the 2^nd^-order dynamics system combined with the target impedance model described in (4) and (8). Assuming a constant virtual mass with time-varying virtual spring and damper and considering the following positive definite Lyapunov function

(30) $$\begin{array}{c} V=\frac{1}{2}m_{d}{\left(\dot{x}-{\dot{x}}_{r}\right)}^{2}+\frac{1}{2}k_{d}\left(t\right){\left(x-x_{r}\right)}^{2}. \end{array}$$

Taking the derivative of the last equation and substituting (8) into (30) lead to

(31) $$\begin{array}{c} \dot{V}=f_{e}\left(\dot{x}-{\dot{x}}_{r}\right)+\left(\frac{1}{2}{\dot{k}}_{d}\left(t\right){\left(x-x_{r}\right)}^{2}-b_{d}\left(t\right){\left(\dot{x}-{\dot{x}}_{r}\right)}^{2}\right). \end{array}$$

According to the last equation, the time-varying virtual stiffness could violate the passivity condition, whereas the virtual damping term could have a positive effect on the energy dissipation. However, assuming constant-value virtual parameters can ensure the system stability as follows.

(32) $$\begin{array}{c} \dot{V}=f_{e}\left(\dot{x}-{\dot{x}}_{r}\right)-b_{d}{\left(\dot{x}-{\dot{x}}_{r}\right)}^{2}\leq f_{e}\left(\dot{x}-{\dot{x}}_{r}\right). \end{array}$$

Integrating the last equation to get the following satisfied passivity condition gets

(33) $$\begin{array}{c} V\left(t\right)-V\left(0\right)\leq \int_{0}^{t}f_{e}\left(\tau \right)\;\left(\dot{x}\left(\tau \right)-{\dot{x}}_{r}\left(\tau \right)\right)d\tau . \end{array}$$

See [43] for more details on passivity conditions for variable impedance control.

**Remark 2 .**

It is important to keep in mind that the time-varying virtual stiffness parameter can be the critical determinant of the system stability. Literature proves that there are two options for time-varying virtual impedance mass/inertia: (1) it can be of constant value with no effect of Lyapunov stability or (2) it can be of a value equal to the mass/inertia of the robot end effector. The last case can be exploited to design a control law free of contact force feedback; see [43, 73] and the references therein. In addition, the virtual damping parameters can be selected as $d\left(t\right)=2\xi \sqrt{m\;k\left(t\right)}$, where *ξ* > 0 is a constant damping ratio.

In effect, four techniques are possible to deal with active variable impedance control:

- manipulation of the virtual stiffness term such that it is related to interaction force error via a PID family controller; see the work of [62, 69, 79] described in the last section. However, this strategy assumed time-varying virtual stiffness only with other constant-value impedance parameters
- neglecting the virtual stiffness term of the impedance model and manipulating the virtual mass and damping terms. Tsumugiwa et al. [80] proposed variable impedance control for human-robot cooperative calligraphy with a time-varying virtual damping term only. The idea is to adjust the target-damping coefficient of the robot impedance function proportional to the estimation of the arm stiffness of the human operator. This procedure may avoid instability due to increased stiffness of the operator's arm. Even if the stiffness of the human operator's hand is very high, introducing a low damping coefficient for the target impedance of the robot may lead to stable operation. Ficuciello et al. [73] improved the performance of impedance control of a 7-DoF KUKA LWR4 by exploiting the kinematic redundancy and modulation of impedance parameters (the virtual mass and damping terms) such that they imitate human behavior. The authors found that redundancy may enlarge the stability margins of the impedance parameters. In addition, the virtual variable impedance behavior with convenient modulation of the time-varying parameters might be superior compared to constant coefficient impedance behavior. The variable impedance target may (i) enhance the performance and safety of the interaction tasks with humans and (ii) make a compromise between accuracy and execution time
- augmentation of the impedance model with an energy-storing element whose role is to store the energy dissipated by the controlled system such that the passivity conditions are satisfied. With this scheme, impedance control with time-varying stiffness matrices can be a powerful tool to deal with a compliant environment that requires time-varying interaction forces. This technique has been called energy tank-based impedance control and implemented by [43, 81]. Although the strategy of tank-based impedance control for generating stable interaction forces with variable stiffness is strong [43, 81, 82], it is dependent on the states of the system, which means that it should be applied online
- design of adaptive laws for tracking the virtual damper and spring parameters. However, this technique could impose constraints on the values of the virtual damping and stiffness in order to ensure the system stability; for more details, see the work of [83]. In addition, Kronander and Billard [19] found that the admissible stiffness profile could depend on the states of the robot that can be unknown beforehand. Therefore, they proposed a state-independent scheme to ensure stability of variable impedance control. This means that the time-varying parameters (damping and stiffness) of the target impedance behavior can be applied offline before the performance of the task

However, some works injected the time-varying stiffness directly to the impedance model without considering the overall system stability and the associated passivity conditions, e.g., see [84]. In general, the following points need to be considered:

- There are two strategies for imitation of human impedance behavior: variable impedance actuators [20, 85–88] and active impedance control with suitable modulation for the time-varying tuned parameters
- A challenge in the application of variable impedance target with human behavior is how to transfer the impedance characteristics from humans to robots and ensure overall system stability. Various techniques are available for estimation of human impedance: most are based on neurological schemes, such as the human central nervous system [89], learning control strategy [90], and teleimpedance [91]. On the other hand, the Lyapunov theory is a powerful tool to investigate the validity of the proposed impedance model. In addition, a systematic tuning method is proposed in [92]
- In general, if the robot is to be freely driven by the human, robot impedance should be low; zero stiffness is recommended in this case. For fast motion purposes, virtual robot damping should be decreased and vice versa, whereas decreasing virtual inertia may lead to instability problems [73]
- On the other hand, regulation of the virtual stiffness of the host robot is necessary for surgery, rehabilitation applications, and collaborative robots [73, 93]. Most researchers have focused on manipulation of the system's stiffness due to (1) the wide range of adjustability of the system stiffness compared with damping and inertia coefficients, (2) because the stiffness term is the dominant factor for low-velocity motion, and (3) because the stiffness term has a considerable effect on system stability for the steady-state regions. For details on the stiffness of human limbs, see [94–97]. However, the time-varying virtual stiffness should be associated with guaranteed stability
- With constant parameter-based impedance control, the passivity property is conserved; however, with arbitrary time-varying parameters for impedance behavior, the passivity property can be lost [19]

## 5. Active Impedance Control of Constant Impedance Flexible Joints

**Figure 7:**
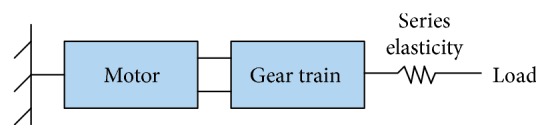
Simplified schematic for series elastic actuators (SEA) [56].

In this section, impedance control of robots with flexible elements is discussed. The focus is on constant impedance series elastic actuators (SEAs). The cascade control combined with an outer impedance loop is often proposed for these types of compliant actuators; for more details on cascade control theory, see [98–102]. The general structure of a flexible joint can consist of three components: the actuator, the gear train, and the elastic element that may be in series with the output link [20] (Figure 7).

Accordingly, the actuator is called constant SEA or variable stiffness actuator (VSA) based on the behavior of the designed joint stiffness (constant stiffness or variable). In this category of actuators, the actuator does not control the link directly but will exchange energy with the transmission system that generates the flexible torque that actuates the link. In recent technology, flexible joints are integrated with robots to guarantee safe motion during the contact phase or to attenuate the impact shock of unexpected forces [103, 104]. The classical rigid body formulation for robots may be inadequate for motion in complicated tasks. The flexibility may exist due to the compliance of the gear transmission, belts, and drive shafts. Adding the elastic element in series with the actuator and the output load can have the following characteristics [105–108]:

- It serves as an accurate torque source and as a low-cost torque sensor
- The elastic element also serves as a compliant interface between the human and the robot, protecting the user and actuating system from sudden shocks and improving back drivability characteristics. In effect, the contact force can be indirectly regulated and controlled by the passive elements
- The motor is isolated from shock loads, and hence, the dynamic effects of backlash and friction can be filtered by the flexible element
- A drawback is the reduced large torque bandwidth due to motor saturation

The following points need to be considered when designing flexible joints:

- The output flexible torque is important in the performance of interaction tasks; the flexible element should be exploited in the control structure rather than dealing with it as a disturbance source
- The flexible element (e.g., spring) acts as a force sensor allowing the actuator output force to be controlled, and hence, the design of the control law could be easy; see [105] for more details
- The behavior of the flexible transmission cannot be known completely for variable impedance actuators, due to the inherent nonlinearity and associated complexity

Recalling (3), the Lagrangian formulation for robots with flexible joints (e.g., simple harmonic drive, SEA, or even VSA) in constrained space can be expressed as [109, 110]

(34) $$\begin{array}{c} M\left(q\right)\;\ddot{q}+C\left(q,\dot{q}\right)\dot{q}+g\left(q\right)=J{\left(q\right)}^{T}f_{e}+\tau_{s},\\ I_{m}\ddot{\theta }+\tau_{f}\left(\dot{\theta }\right)+G\tau_{s}=\tau_{m},\\ \tau_{s}=K_{s}\left(G\theta -q\right),\\ L\dot{ı}+Rı+K_{b}\dot{\theta }=u, \end{array}$$

where *K*_*s*_ is the spring stiffness matrix and the other nomenclatures are defined previously.

According to (34), some major problems can be produced due to the presence of the joint flexibility, e.g., (i) the degree of freedom of the robotic system increases twofold, (ii) the resulting system is not fully actuated due to the induced joint flexibility, (iii) the joint flexibility results in fast dynamics which may stimulate the vibration problems, and (iv) for motion in constrained space, a small deviation in joint position may lead to excessive contact force on the environment due to the coupling effect [111, 112] (The dynamic model of a flexible joint-actuated robotic system can be considered as a slow-fast system (i.e., a system with different time scales). The modal frequencies associated with the fast dynamics are well separated from the rigid body modes. This assumption is associated with the singular perturbation theory; see [113, 114] for more details).

Different techniques are available to deal with the control of flexible joints: decoupling control schemes [111, 115, 116], backstepping control [117], singular perturbation control [118], and adaptive control [57, 119–121]. Efforts have been performed for controlling arbitrary stiffness flexible-joint robots in free space [122–124] and in constrained space [40, 57, 59, 112]. The joint torque control is essential for vibration damping during the free space motion and soft and safe interaction control during the contact phase [125]. Modifications of (34) to meet the requirements of inner torque control may require calculation of the fourth derivatives of the angular positions and measurement of the derivatives of a torque sensor (which could be rather noisy) [112] (Elastic joints usually avoid torque sensors by measuring the spring displacement. Such solution usually leads to a clean force derivative [125].). In effect, (34) should be modified such that the full dynamics has output variables (*q*, *τ*_*s*_) with the input control *u*. Albu-Schäffer et al. [125] described a passivity-based impedance control framework based on motor position and joint torque signals, as well as their first-order derivatives. It provides a high degree of robustness to unmodeled robot dynamics and in the contact with unknown environments. The proposed control law consists of two terms: (1) the first term is to regulate the joint-level impedance and (2) the second term is a torque feedback loop. Second- and higher-order derivatives of the variable states are not required, which give some preference to other techniques that use third derivatives of the variable states; see [29] for more improvements.

**Figure 8:**
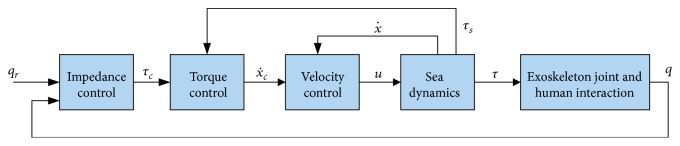
Multilevel control of the LOPES robotic system where the subscript *c* refers to the commanded signals [107].

The rest of this section considers specifically the possible problems associated with nested control loops of the SEA. Many researchers used simple force control of SEA-driven robots based on linear control theory. However, the system stability and passivity conditions should be satisfied in order to achieve feasible performance [126, 127]. A comprehensive overview of existing controller passivity is presented in [128]. Pratt et al. [129] have proposed a passive force control architecture including some feedforward terms and one PID-based feedback for tracking the desired interaction force. The authors replaced the integral term of the PID controller by a first-order low pass filter for ensuring passivity but with possible static errors. Yuan and Stepanenko [122] and Lozano and Brogliato [123] proposed to use an inner velocity loop combined with an outer force control to improve the system performance and overcome some undesired effects of the actuator and the transmission element. For robots with SEAs, cascade control is often implemented with an outer impedance loop and inner torque control. In effect, there are miscellaneous nested loops coupled with the impedance control (see details below). Currently, torque sensors are widely available; however, using an inner torque loop combined with another control loop can encounter challenges associated with stability problems. For example, the PI-based torque control might be difficult to tune or to provide a high bandwidth if the load side has some damping. In addition, the control feedback might require a torque sensor, which is always noisy [56]. Vallery et al. [126, 127] described cascade control with three nested loops: the outer impedance loop for regulating the relationship between the interaction force and the reference trajectory, an inner torque control, and the innermost velocity loop. The passivity conditions for the rendering of a pure spring are derived, and the control gains are selected. In effect, the same structure for impedance control of SEA has been proposed in [107, 130]; see Figure 8.

**Figure 9:**
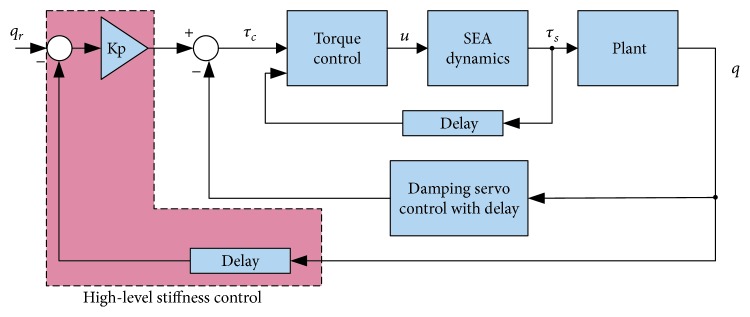
Proposed cascade control with three control loops: high-level stiffness servo loop, inner embedded damping servo loop, and the innermost torque control [131].

Tagliamonte et al. [130] investigated the performance and stability conditions for three multilevel control loops: an outer impedance loop with a virtual spring-damper system, and an inner PI torque control generating a set-point reference for the innermost PI velocity loop. The authors proposed guidelines for tuning the controller gains and the possible ranges of virtual impedance parameters based on the passivity theory, generalizing the results of [126]. Zhao et al. [131] proposed a critically damped fourth-order system gain selection criterion for a cascaded SEA control structure with inner torque and outer impedance feedback loops. Velocity filtering and feedback delays are taken into consideration for stability and impedance performance analysis. The proposed scheme is depicted in Figure 9. The authors focused on maximizing the impedance range of SEA because most studies are concerned with low or near-zero impedance dynamics [132, 133]. The authors show that decreasing the impedance gain may lead to instability problems. This is also confirmed in the literature [128]. However, there is no clear result regarding the increase of the inner torque loop gain and its effect on the system stability [134–136].

Mosadeghzad et al. [56] investigated the passivity and implementation problems associated with different control loops (inner position control, inner torque control, and inner velocity control). The authors made important observations as follows.

- The discrete impedance control system may require the lower bounds for the bandwidth of the inner control loop; however, in a continuous time control system, larger values for the inner loop gains can be obtained to ensure the stability and achieve high overall bandwidth
- With the inner torque control loop, the model of the host robot should be known to avoid instability. However, using only an inner torque loop, the system can drift and this is because torque control only stabilizes the torque response of the system but does not provide internal stability. This can be overcome by setting an outer loop with PD control, a zero reference position, and velocity and even setting the *P* gain to zero but using a nonzero *D* gain to avoid drift. This still does not control position but avoids drift in the system. The use of an inner position control avoids these problems, but if a controller with integral action is used, then passivity is greatly impaired and there might be interactions with passive environments that cause instability

Li et al. [137, 138] proposed an adaptive MIMO human-robot interaction control for a SEA-actuated robot. An adaptive single controller was proposed to deal with the two motion modes associated with the human-robot interaction: robot-in-charge mode and human-in-charge mode. It is a two-level control architecture: high-level control for designing force region function-based virtually desired joint references and low-level control for tracking control of the desired references for the SEA considering the uncertainty of the system. (The force region function is used to monitor the variation of the interaction force, and hence, based on it, a weight factor is designed. The adjustment of the target impedance can be achieved by manipulating the weight factor.)

In view of the above statement, the following points can be noted:

- The control architecture of the SEA can consist of three nested control loops: an innermost velocity loop, an intermediate torque loop, and the outer impedance control loop that renders virtual impedance for safe and comfortable human-robot interactions: see [107, 126, 127, 130, 134]. However, many control schemes do not use the innermost velocity loop [139–145]. In addition, a nested loop using acceleration feedback has been proposed showing excellent results in terms of both performance and stability [105, 146]
- A powerful tool for tuning the gains of cascade control of the SEA and determining the ranges of the virtual impedance target is the passivity theory. Most work has focused on stability/passivity constraints of cascade control for single SEA-actuated joints using linear control theory. Extending the work for MIMO robots considering the nonlinearities, time delay problems, and stability problems associated with the coupling nested loops is not straightforward; see [138] for example. In addition, the control algorithms that regard arbitrary stiffness joint can be exploited for motion stabilization of a SEA-actuated robot
- For pure virtual spring impedance target, the maximum value of the virtual spring impedance can overcome the physical stiffness while retaining passivity [128]

**Remark 3 .**

1. Most of the standard control schemes of soft robots such as high-gain robust/adaptive control, feedback linearization, and active impedance control attempt to regulate/control the target system at the expense of stiffening it. Therefore, Santina et al. [147] showed that using a feedforward control loop combined with a low-gain feedback gain can achieve better-desired behavior comparing with feedback control schemes
2. The constant impedance actuators described above may have limitations associated with dealing with the different tasks and motion frequencies; the different tasks need variable stiffness (impedance) actions that could be lost in the SEAs. Therefore, robotic systems with VSAs are capable of rejecting disturbances, storing energy, and controlling the end effector stiffness in contact space [41]; see [20–22, 148] for details on the design, performance, and classification of VSAs
3. In general, there are three control schemes of VSAs: (i) simultaneous control of position and stiffness control [149], (ii) impedance control with inner torque control [150, 151], and (iii) bioinspired control, e.g., time-based compliance control [152] and coactivation control [153–155]
4. The proposed control schemes, the performance, and stability have not yet been extensively investigated. The stability of impedance control associated with VSAs requires more research. However, impedance control associated with inner torque control is the easiest control scheme to deal with constant and VSAs. For more details on the control architecture and stability of VSAs, see [149–151, 156–169]

## 6. Conclusions

This paper is aimed at systematically introducing the features and limitations of the categories of impedance control schemes. Basically, impedance control can be classified as force-based impedance control and position-based impedance control. The conventional impedance control schemes do not consider the force tracking problems in the outer impedance filter, resulting in a deviation of the desired force references. Accordingly, modification of the impedance filter to satisfy the force-tracking problem is a motivating technique of imitation of human behavior. As mentioned, one strategy for force tracking-based impedance control is to change the virtual stiffness. Therefore, a clear connection is required between variable impedance control and force tracking-based impedance control. On the other hand, changing the impedance parameters is not trivial; investigation of the stability problems of variable impedance control requires additional work.

Impedance control of flexible-joint actuated robots remains a challenge. Control of robots with constant impedance joints could be easier than variable impedance joints. In variable impedance actuators, the stiffness is an added variable output that should carefully be controlled. In general, an outer impedance filter integrated with torque control is an effective strategy to solve this category of transmission. In general, a careful control architecture is required to exploit joint flexibility. For example, using the standard feedback control schemes may make the system stiffer, and hence, the system behavior changes. Therefore, bioinspired-based control systems such as feedforward action can work well to exploit the system impedance.
